# Enhanced Proliferation and Differentiation of Human Osteoblasts by Remotely Controlled Magnetic-Field-Induced Electric Stimulation Using Flexible Substrates

**DOI:** 10.1021/acsami.3c09428

**Published:** 2023-12-05

**Authors:** Oriol Careta, Aliona Nicolenco, Filippos Perdikos, Andreu Blanquer, Elena Ibañez, Eva Pellicer, Christina Stefani, Borja Sepúlveda, Josep Nogués, Jordi Sort, Carme Nogués

**Affiliations:** † Departament de Biologia Cellular, Fisiologia i Immunologia, Universitat Autònoma de Barcelona, Bellaterra, Cerdanyola del Vallès E-08193, Spain; ‡ Departament de Física, Universitat Autònoma de Barcelona, Bellaterra, Cerdanyola del Vallès E-08193, Spain; § CIDETEC, Parque Científico y Tecnológico de Gipuzkoa, Paseo Miramón, 191, San Sebastián 20014, Spain; ∥ Catalan Institute of Nanoscience and Nanotechnology (ICN2), CSIC and BIST, Campus UAB, Bellaterra, Barcelona E-08193, Spain; ⊥ Instituto de Microelectronica de Barcelona (IMB-CNM, CSIC), Campus UAB, Bellaterra, Barcelona E-08193, Spain; # Institució Catalana de Recerca i Estudis Avançats (ICREA), Pg. Lluís Companys 23, Barcelona E-08010, Spain

**Keywords:** magnetoelectric heterostructure, flexible biomaterial, magnetoelectric stimulation, wireless actuation, proliferation, differentiation, osteoblasts

## Abstract

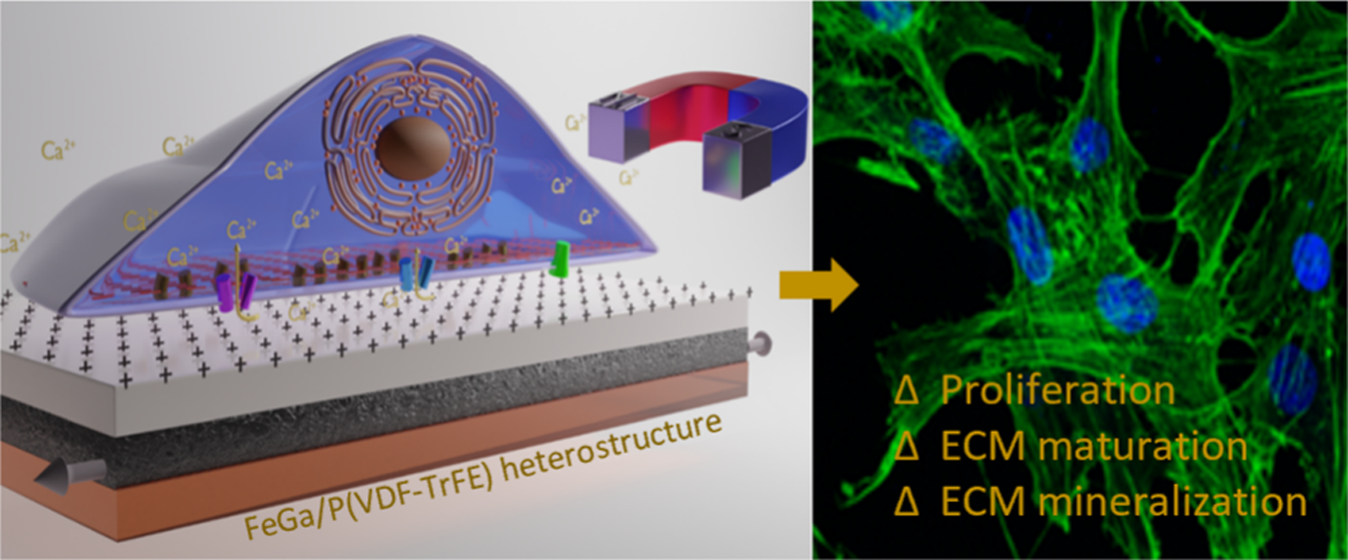

With the progressive aging of the population, bone fractures are an increasing major health concern. Diverse strategies are being studied to reduce the recovery times using nonaggressive treatments. Electrical stimulation (either endogenous or externally applied electric pulses) has been found to be effective in accelerating bone cell proliferation and differentiation. However, the direct insertion of electrodes into tissues can cause undesirable inflammation or infection reactions. As an alternative, magnetoelectric heterostructures (wherein magnetic fields are applied to induce electric polarization) could be used to achieve electric stimulation without the need for implanted electrodes. Here, we develop a magnetoelectric platform based on flexible kapton/FeGa/P(VDF-TrFE) (flexible substrate/magnetostrictive layer/ferroelectric layer) heterostructures for remote magnetic-field-induced electric field stimulation of human osteoblast cells. We show that the use of flexible supports overcomes the clamping effects that typically occur when analogous magnetoelectric structures are grown onto rigid substrates (which preclude strain transfer from the magnetostrictive to the ferroelectric layers). The study of the diverse proliferation and differentiation markers evidence that in all the stages of bone formation (cell proliferation, extracellular matrix maturation, and mineralization), the electrical stimulation of the cells results in a remarkably better performance. The results pave the way for novel strategies for remote cell stimulation based on flexible platforms not only in bone regeneration but also in many other applications where electrical cell stimulation may be beneficial (e.g., neurological diseases or skin regeneration).

## 1 Introduction

Due to the increased aging of the population, bone fractures are a major health concern which causes an increasing economic burden.^1^ To mitigate the diverse effects of bone fractures, reducing the recovery time of patients is fundamental. Hence, diverse strategies to help the healing of fractured bones are being investigated. Applied or induced electric charges have been shown to play a fundamental role in promoting cell differentiation and proliferation.^2−4^ Endogenous currents related to inherent mechanical strains are important for bone remodeling.^2,5^ In fact, during recovery, bones are capable of generating electric potentials that facilitate bone regeneration.^6^ These electric potentials can be naturally induced through piezoelectricity, which is attributed mainly to collagen.^7^ Some authors have suggested that bone endogenous electricity enhances the process of bone healing by the stimulation of the calcium–calmodulin pathway, which induces the upregulation of several promoters of bone regeneration, such as bone morphogenetic proteins, transforming growth factor-β, and other cytokines.^8−10^ Remarkably, application of exogenous electricity in large bone fractures where the bone regeneration is impaired has been demonstrated not only to enhance osteoblast proliferation but also to regulate proteoglycan and collagen synthesis, as well as to accelerate bone formation and repair.^11−13^

Traditionally, electrical stimulation has been applied to cells in vitro studies through direct current stimulation using electrodes either in direct contact with a conductive substrate on top of which the cells are grown or contacting the culture medium. Even though the use of electrodes is the easiest option and the most widely used method, it presents some important drawbacks such as insufficient biocompatibility of the electrodes’ components or the conductive substrates^14^ or hazardous changes induced in the medium such as temperature rise, pH variations, or the generation of harmful byproducts.^15^ In addition, in vivo applications, the use of electrodes can lead to important inflammation effects or even infections.^16^

Because of these disadvantages, efforts have been made to develop energy harvesting materials to induce electrical stimulation without the need of direct insertion of electrodes, but using, instead, the mechanical and chemical energies present in the body.^17^ In this framework, piezoelectric/flexoelectric materials are excellent candidates to assist in this process since they are able to generate electrical voltage in response to mechanical stress.^18,19^ As aforementioned, the bone itself has inherent piezoelectric/flexoelectric properties, producing several electrical and biochemical signals that enhance its growth in response to mechanical activity. Similarly, mechanical stimulation of piezoelectric materials can be achieved either by the application of physiological compressive loads,^20^ by the stress applied by cells growing on the material surface,^21^ or, most commonly, by ultrasounds stimulation.^22^

**Scheme 1:**
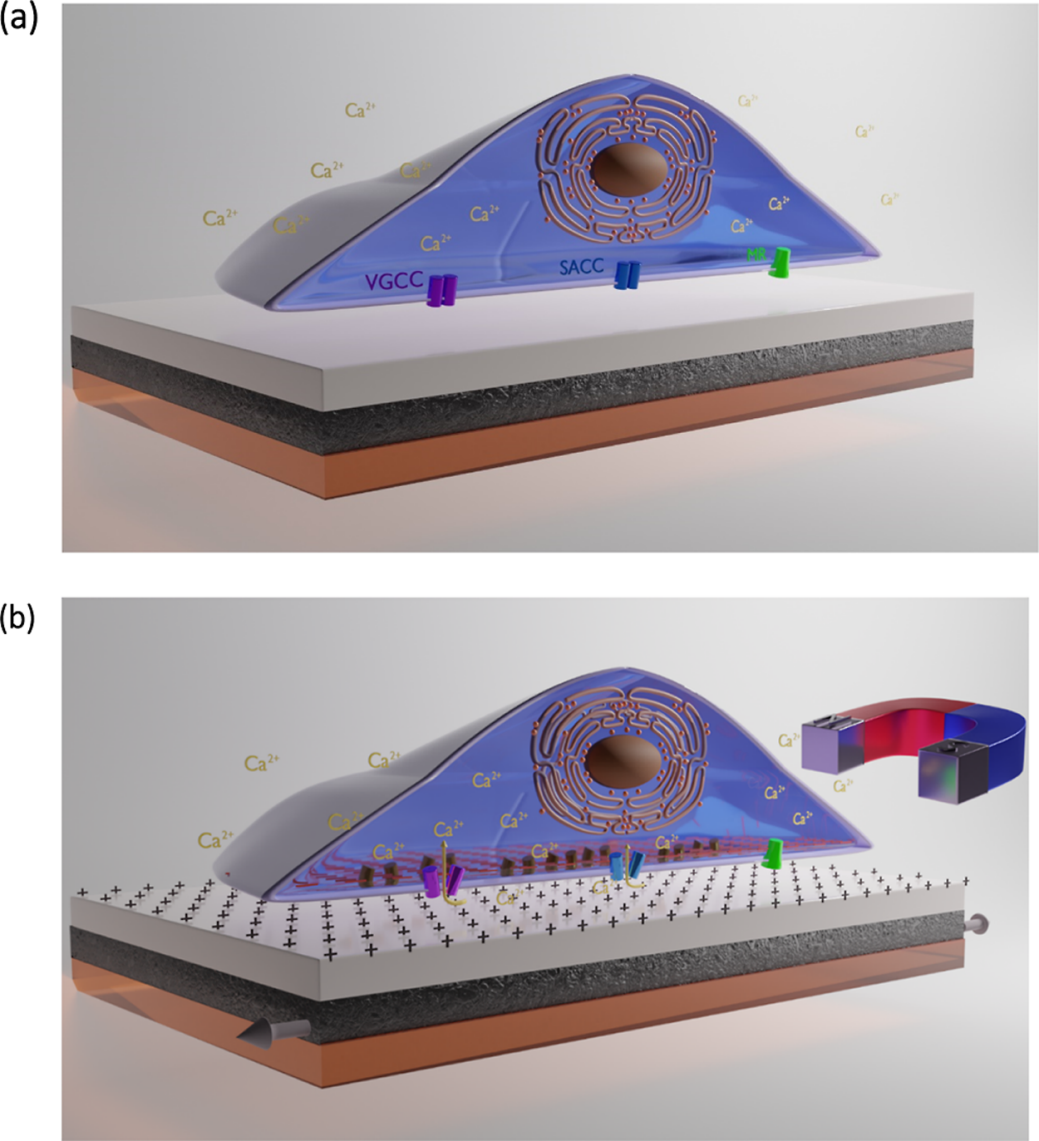
Schematic Representation of the Cell Stimulation Approach by Using Magnetoelectric Materials; (a) Without Magnetic Field, No Electric Field is Induced and, Thus, the Cell is Not Stimulated; (b) When a Magnetic Field is Applied, the Magnetostrictive Layer (Black) Changes Its Shape or It Expands/Contracts, Thus Transferring Strain to the Adjacent Ferroelectric Layer (Gray) in Which Electric Charges (+) Are Created, Thus Generating an Electric Field This electric field stimulates the cells to promote proliferation and differentiation, probably by the opening of the voltage-gated calcium channels (VGCCs) and the stretch-activated calcium channels (SACCs), which allow for the inward flow of Ca^2+^ ions. This is possible when a flexible substrate is used (in this case, kapton, depicted in orange color). MR: membrane receptor.

Even though the use of piezoelectric materials stimulated by the cells growing on their surface represents a great improvement compared with traditional electrode stimulation (i.e., avoiding direct contact between cells and electrodes), the fact that both the generated electrical potential and the application time cannot be controlled constitutes a drawback for their use. For this reason, new ways to mechanically stimulate piezoelectric materials remotely in a more controllable way, such as using ultrasounds, are being developed. However, ultrasounds are known to have several limitations for their use on biological tissues, such as limited penetration, local temperature increase (which can be particularly significant in bones), cavitation, or acoustic streaming, just to mention a few.^23,24^ These can result in cell detachment, changes in membrane permeability, or even cell lysis. Additionally, the frequencies used in ultrasounds experiments are exceedingly high (in the MHz range, i.e., ns–μs stimulation) for the typical response of cells (which commonly occurs in the ms range).^25^ Thus, in recent years, alternative stimulation pathways are being explored.^26^ Among them, magnetoelectric actuation,^27−30^ which uses rather low intensity, highly penetrating, magnetic fields at low frequencies, is emerging, with stimulation times that are commensurate with the cell response. Magnetoelectric materials are materials in which electric polarization can be generated by magnetic fields or, conversely, magnetization can be modulated with electric fields. A common approach to this type of materials is to combine a ferromagnetic magnetostrictive layer with a ferroelectric or piezoelectric layer.^31,32^ When these structures are exposed to a magnetic field, electric polarization is generated by the strain induced in the magnetostrictive material, which is transferred to the adjacent ferroelectric layer (see Scheme 1). More specifically, when subject to an applied magnetic field, the magnetostrictive layer deforms because of the magnetostrictive effect. This change in shape is transferred to the adjacent ferroelectric layer since they are both mechanically coupled. In turn, the change of shape of the ferroelectric layer induces an electric polarization due to the ferroelectric effect.

Nonetheless, the performance of this type of systems is often limited by clamping effects, i.e., the expansion-contraction constraints imposed by the rigid substrates on top of which these structures are typically grown.^33,34^ As a consequence, when growing magnetoelectric heterostructures onto rigid substrates, the effects are small even when using high magnetic fields, high frequencies, and very long stimulation times. Diverse approaches are being developed to reduce clamping effects, such as the implementation of 3D magnetoelectric structures or the use of magnetoelectric membranes.^35^ However, so far, magnetoelectric systems have demonstrated only a moderate improvement in bone regeneration experiments. Thus, novel approaches to improve the efficiency of bone cell proliferation and differentiation are highly desirable.

Here, we present the use of a flexible magnetoelectric heterostructure [kapton/FeGa/P(VDF-TrFE)], which is able to produce an electrical response upon application of alternating magnetic fields. This magnetoelectric stimulation is efficient in remotely stimulating osteoblasts in vitro (Scheme 1).

## 2 Results and Discussion

The material under investigation consists of a flexible kapton substrate on which a magnetostrictive FeGa layer is grown. The top layer of the magnetoelectric heterostructure is a ferroelectric polyvinylidenefluoride-*co*-trifluoroethylene [P(VDF-TrFE)] layer (see Materials and Methods). The growth of adjacent ferromagnetic/ferroelectric layers in direct contact between each other allows the whole deformation of the magnetostrictive layer induced by the magnetic field to be transferred to the ferroelectric layer, consequently maximizing the generated electric field. Concerning the choice of materials, FeGa was selected as the magnetostrictive layer for several reasons. First, FeGa has one of the largest magnetostrictive constants of rare-earth-free magnetostrictive materials. Moreover, it is easy to grow and is quite resistant to oxidation. In addition, prior investigations within our research group have confirmed its noncytotoxic nature.^36^ These combined properties make it ideal for biomedical applications. The choice of ferroelectric/piezoelectric material [i.e., P(VDF-TrFE)] was supported by its strong ferroelectric response despite being flexible and lead-free. Moreover, the cytocompatibility of P(VDF-TrFE) has been demonstrated in previous studies.^37,38^ Finally, the use of kapton is a keystone of our study, since contrarily to most of the substrates used in magnetoelectric cell stimulation (e.g., silicon), it is rather flexible (see Figure S1; with a Young’s modulus of about 2.5 GPa). The flexibility of the kapton substrate was expected to improve the efficiency of the system compared with analogous heterostructures grown on rigid substrates (with strong clamping effects).

### 2.1 Characterization of the FeGa/P(VDF-TrFE) Heterostructures

**Figure 1:**
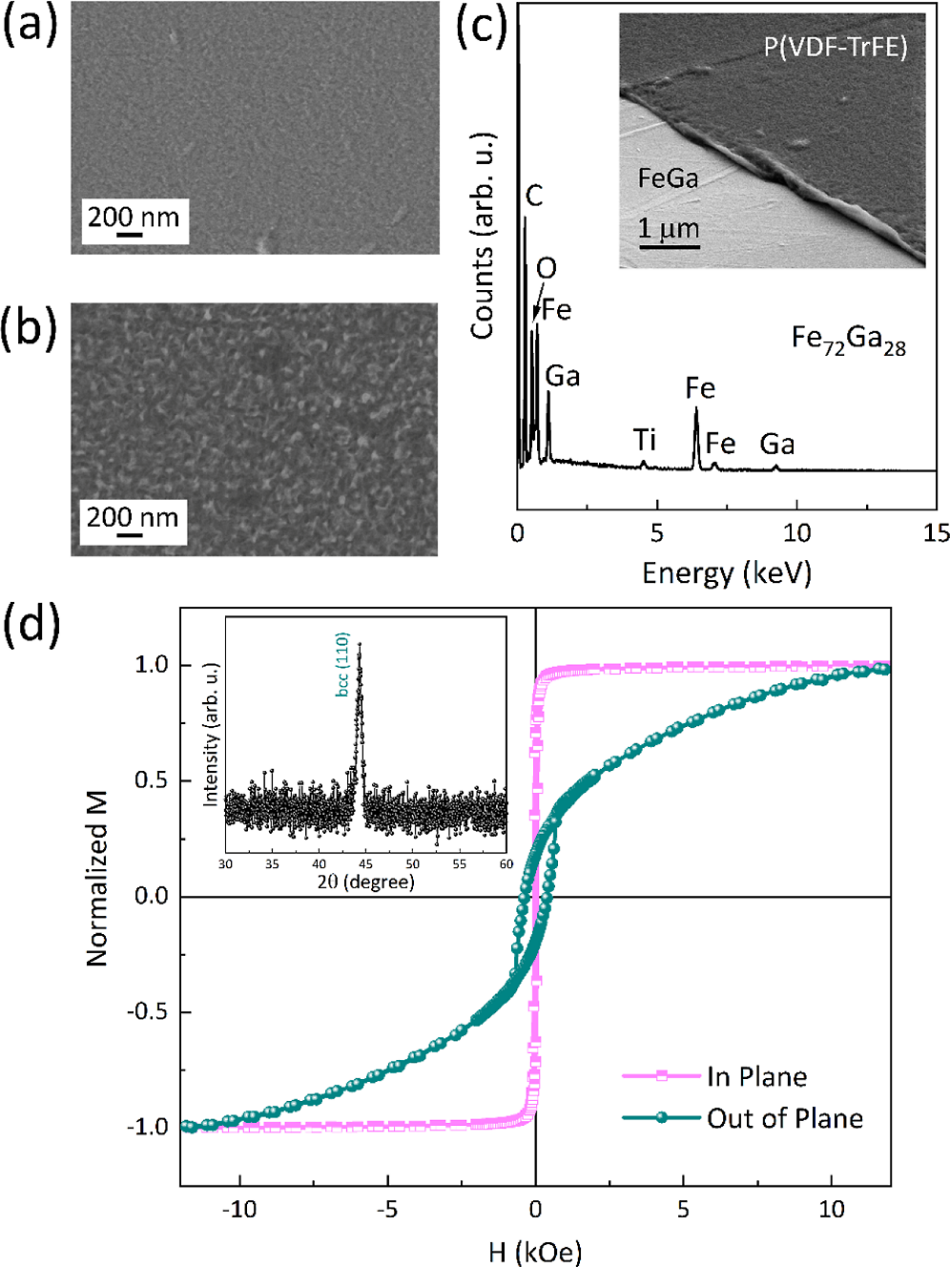
(a) SEM image of the sputtered FeGa films; (b) SEM image of the top P(VDF-TrFE) layer; (c) SEM image taken on one edge of the sample showing the P(VDF-TrFE) layer deposited on top of FeGa (the sample was slightly tilted on purpose to provide the perspective) together with a representative EDX analysis to assess the composition of the FeGa films; and (d) room-temperature magnetic hysteresis loops applying the magnetic field along in-plane and perpendicular (out of plane) directions of the film. The inset in (d) shows the XRD pattern of the FeGa film.

The scanning electron microscopy (SEM) image of the sputtered FeGa layer shows its polycrystalline structure (Figure 1a). The energy-dispersive X-ray spectroscopy (EDX) analysis reveals that the Fe/Ga atomic ratio is 72:28 (±5%) (Figure 1c, i.e., close to the alloy composition that is known to exhibit the highest magnetostriction coefficient for this alloy^39^). The X-ray diffraction (XRD) pattern (inset in Figure 1d) indicates that the FeGa film grows in the expected body centered cubic (bcc) structure, and it is textured along the (110) direction. No evidence for phase separation (i.e., occurrence of Fe-rich or Ga-rich phases) or Fe/Ga oxides was observed by XRD. The magnetic characterization of the films (Figure 1d) reveals a clear in-plane effective magnetic shape anisotropy, with a very square and low-coercivity in-plane hysteresis loop and a tilted out-of-plane loop with larger coercivity. Such coercivity increase in the out-of-plane hysteresis loops has been observed by several authors in FeGa layers grown by different methods, and it has been ascribed to the effects of competing magnetic anisotropies.^40,41^ The SEM image of the P(VDF-TrFE) film grown onto FeGa can be seen in Figure 1b,c. The layer grows homogeneously on FeGa and is clearly polycrystalline with a grain size larger than that of FeGa.

**Figure 2:**
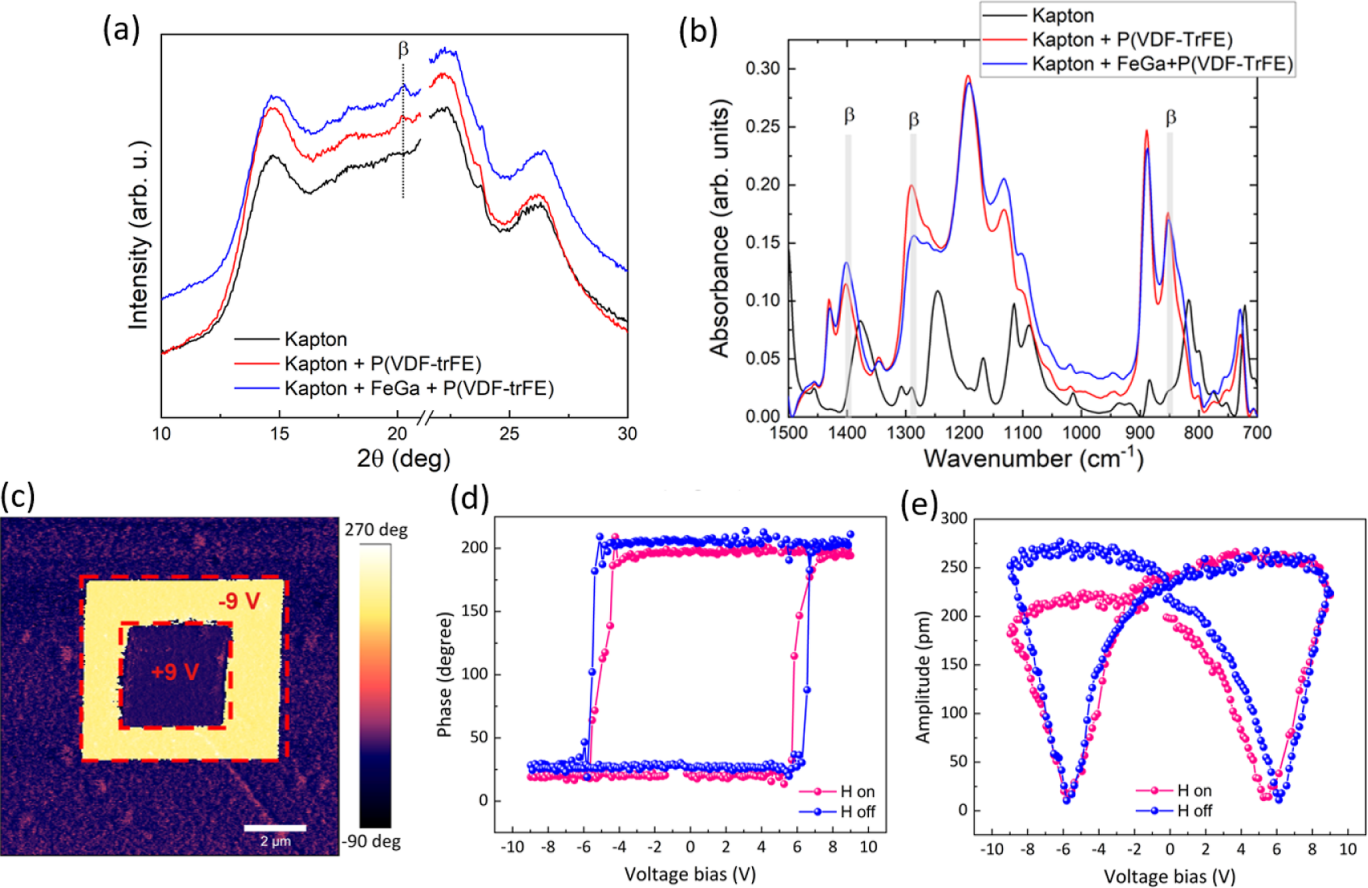
(a) XRD patterns of the P(VDF-TrFE) films grown onto kapton/FeGa (blue curve) and onto kapton (red curve)—the black curve is the XRD pattern corresponding to bare kapton with no layers grown on top (the cut in the 2θ axis is to avoid a peak from the sample holder); (b) FTIR experiments of the same samples as in panel (a), where the peaks shadowed in gray univocally correspond to the β-phase of P(VDF-TrFE); (c) PFM phase image obtained after writing two concentric squares (ferroelectric domains) with opposite polarity, with an applied ±9 DC voltage; and (d) local piezo-response phase and (e) amplitude loops obtained from the FeGa/P(VDF-TrFE) heterostructured films without (H off) and with (H on) an external magnetic field of 1000 Oe, applied along the in-plane direction of the sample.

The XRD patterns of the P(VDF-TrFE) spin-coated onto kapton/FeGa are shown in Figure 2a (blue curve) together with that of bare kapton with no layers on top (black curve) and P(VDF-TrFE) grown onto the kapton substrate using the same growth conditions (red curve). The peak at 2θ = 20.1° corresponds to the (110)/(200) reflections of the β-phase of P(VDF-TrFE),^42^ and it is superimposed to the kapton diffraction background.^43^ Importantly, the β-phase of P(VDF-TrFE) is the one showing optimum ferroelectric properties.^44−47^ Further evidence for the formation of the β-phase is obtained by Fourier transform infrared (FTIR) measurements^48^ (shown in Figure 2b). Note that the inclusion of the FeGa layer has no effect on the formation of the β-phase.

### 2.2 Ferroelectric Properties of the FeGa/P(VDF-TrFE) Heterostructures

The ferroelectric properties of P(VDF-TrFE) were assessed by piezo-response force microscopy (PFM). The layer was prepoled by applying a constant DC bias through the tip of −9 V in a 5 × 5 μm^2^ squared area. After the frame was completed, a consecutive, concentric square of 2.5 × 2.5 μm^2^ was performed with a constant DC bias of the opposite magnitude, +9 V (Figure 2c). The ability to switch the ferroelectric polarization by applying external electric fields leading to stable domains after electric field is switched off demonstrates the good ferroelectric properties of P(VDF-TrFE). Further evidence of ferroelectricity is obtained from the phase versus voltage bias loop (Figure 2d), where an electric coercive voltage of around 6 V is observed (hence smaller than the DC bias strength used to switch the polarization in Figure 2c). In addition, the amplitude versus bias curve shows the typical “butterfly” shape dependence of a ferroelectric material (Figure 2e)^44^ Note that the phase switching angle is close to 180°, indicating a major contribution of the electromechanical response over the electrostatic one. The asymmetry of the butterfly loops might originate from different factors such as a dissimilar work function at the bottom (FeGa) and top (PtIr-coated Si probe) electrodes or occurrence of internal electric field distribution across the P(VDF-TrFE) layer.^49^

### 2.3 Magnetoelectric Coupling of FeGa/P(VDF-TrFE) Layers

Next, the magnetoelectric coupling between the FeGa and P(VDF-TrFE) layers was directly probed by PFM at a given location of the sample, comparing the phase and amplitude curves without a magnetic field and with an applied in-plane magnetic field of 1000 Oe. Note that this magnetic field is sufficient to magnetically saturate the FeGa layer, thus ensuring a significant effect of magnetostriction on the eventual strain-mediated magnetoelectric coupling. In the absence of external magnetic field, the coercive voltages for the FeGa/P(VDF-TrFE) heterostructure are −5.8 and +6.6 V, giving an average coercive electric field of 62 MV m^–1^, which is a typical value for P(VDF-TrFE).^47^ Application of the magnetic field results in an overall decrease of the coercive electric field to a value around 55 MV m^–1^. This reduction of the coercive electric field is in agreement with previous results from the literature^44^ and it indicates a decrease of the energy barrier to switch the ferroelectric polarization. This suggests that the induced magnetostrictive strain in the FeGa film is transferred to P(VDF-TrFE), facilitating voltage-driven reorientation of the ferroelectric domains. In other words, a net electric field is generated in the system by the application of external magnetic fields (direct magnetoelectric effect). The applied magnetic field also causes a change in the ferroelectric amplitude versus voltage butterfly loops, which is another sign of magnetoelectric coupling. One way to estimate the local magnetoelectric coupling coefficient is to assess the change in the asymmetry of the piezo-response loops when the magnetic field is applied, i.e., α_E_ = Δ*E*/Δ*H*, where Δ*H* is the increment in the applied magnetic field and Δ*E* the induced change in electric coercive voltage.^49^ Using this method, the local magnetoelectric coefficient in our case is 50 V cm^–1^ Oe^–1^. This value is comparable to other magnetoelectric heterostructures using P(VDF-TrFE) as their ferroelectric counterpart.^50^ This quantification should be taken with some caution. Namely, since PFM is a local technique, the electric field generated by the tip is very inhomogeneous and it cannot be easily quantified, since it depends on many factors (e.g., the shape of the tip, the conductivity of the layers, the roughness of the films, among others).

### 2.4 Wettability Characterization

**Figure 3:**
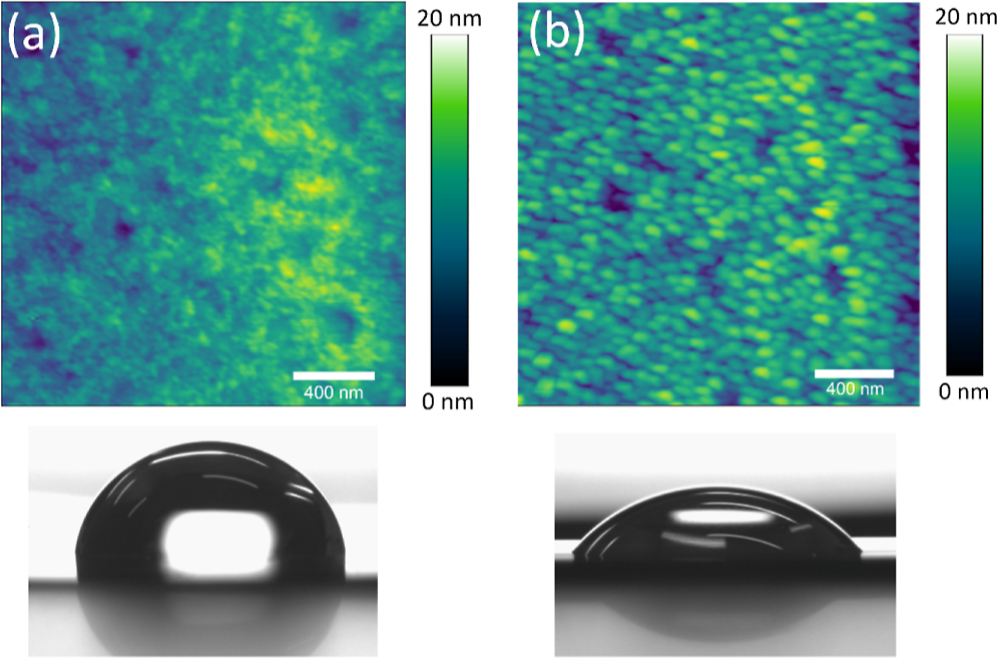
AFM topological images (top) and contact angle measurements (bottom) of (a) the as-grown and (b) the oxygen plasma-treated FeGa/P(VDF-TrFE) heterostructures.

To facilitate cell adhesion and proliferation, it is important to have hydrophilic surfaces; otherwise, cells easily detach and die during their growth. One of the drawbacks of P(VDF-TrFE) and other similar ferroelectric polymers is that they are hydrophobic.^51,52^ This is evidenced in Figure 3, where a contact angle of around 90° is obtained for the as-grown (i.e., untreated) heterostructure. Interestingly, the contact angle significantly decreases to 40° after exposure of the outer P(VDF-TrFE) layer to an oxygen plasma treatment. Topological images obtained by atomic force microscopy (AFM) indicate that oxygen plasma induces some changes in the surface topography. In particular, the P(VDF-TrFE) grains become more visible, although the overall surface roughness remains below 20 nm for both the as-grown and treated samples. When the P(VDF-TrFE) is exposed to oxygen plasma, highly reactive oxygen species are generated, resulting in the formation of oxygen-containing functional groups on the surface which enhance the hydrophilic character of the material.^37^

### 2.5 Effect of the Magnetic-Field-Induced Electrical Stimulation on Osteoblast Viability, Morphology, and Adhesion

Human osteoblast cells (hOBs) were seeded on top of the magnetoelectric heterostructures and kept under standard culture conditions for 24 h to allow cell adhesion. Then, the heterostructures with the adhered hOBs were transferred to the cuvettes of the magnetic stimulation setup (see Materials and Methods) and cultured daily either under a magnetic field of 400 Oe@100 Hz for 1 h to induce an electrical stimulation (electrically stimulated; ES) or without a magnetic field (not electrically stimulated; n-ES).

**Figure 4:**
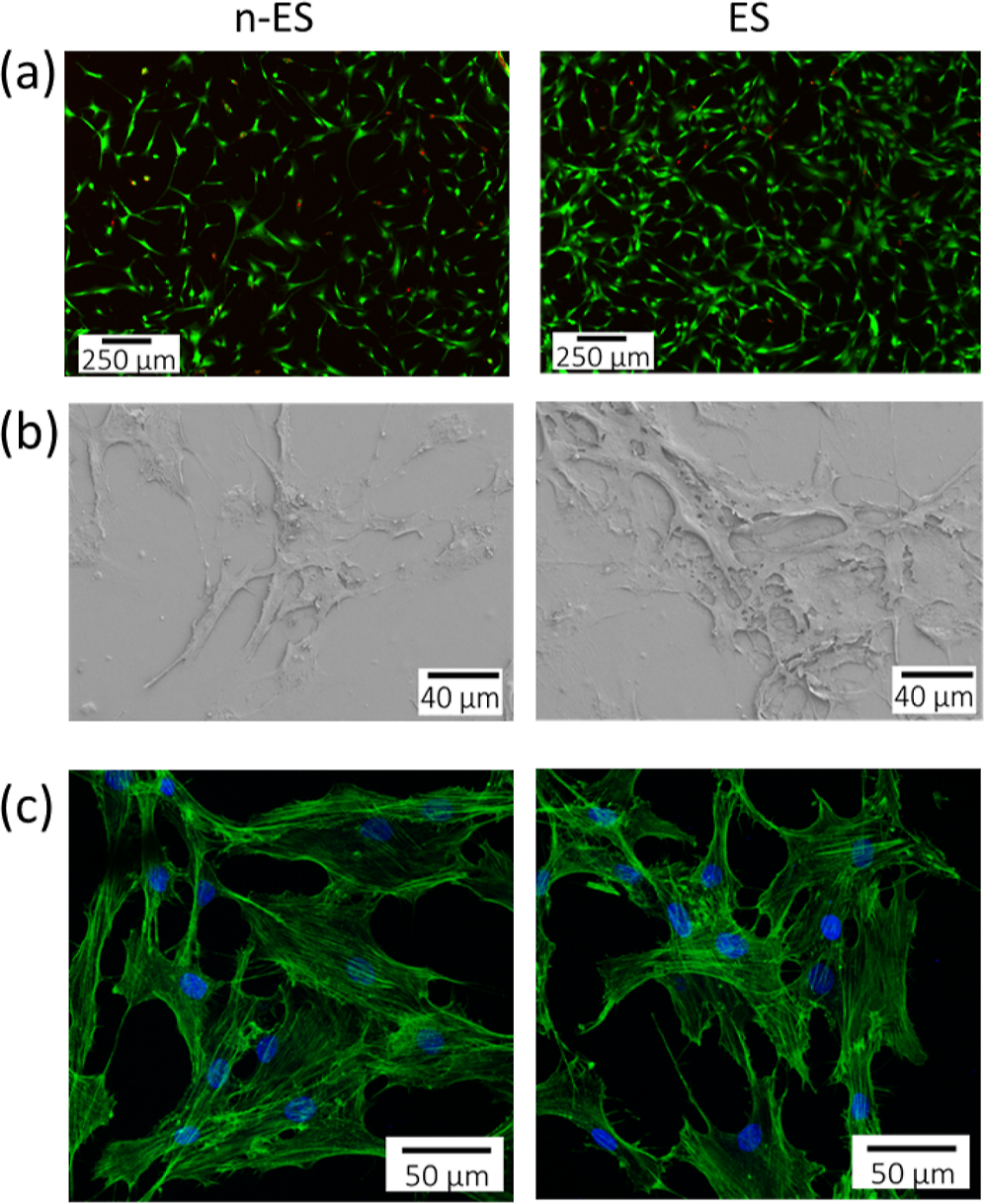
Viability, morphology, and adhesion of osteoblasts cultured on top of the FeGa/P(VDF-TrFE) heterostructures either under magnetic-field-induced electrical stimulation (ES) or without stimulation (n-ES). (a) Cell viability of osteoblasts after 3 days in culture. Live and dead cells appear in green and red, respectively. (b) SEM images of the osteoblasts cultured under ES and n-ES conditions for 3 days. (c) Cytoskeleton (actin, green) and nuclei (blue) of osteoblasts cultured in ES and n-ES conditions for 3 days.

A live/dead kit was used to determine the viability of the cells grown on the surface of the heterostructures. As shown in Figure 4a, cells were able to grow on the heterostructures in both conditions (n-ES and ES). In both cases, a high number of live cells were observed after 3 days in culture (95 ± 3% and 97 ± 3% of live cells for the n-ES and ES conditions, respectively). Nonetheless, the images revealed a significantly higher number of cells on top of the heterostructures under magnetoelectric stimulation (32,000 ± 5000 cells/heterostructure) than without applying the magnetic field (14,000 ± 3000 cells/heterostructure).

The magnetoelectric heterostructure showed remarkable cytocompatibility. Neither the P(PVDF-TrFE) component nor the FeGa or kapton materials exhibit any toxicity toward the cells. This outcome aligns with other studies, where P(VDF-TrFE),^37,38^ FeGa,^36,53,54^ and kapton^55,56^ have been shown to have no adverse effects on cell proliferation.

After the viability assessment, cell morphology was studied through SEM analysis of hOBs grown on the heterostructures under the two conditions. In both cases, cells were evenly distributed on the surface after 3 days of culture (Figure 4b). Overall, hOBs under ES and n-ES conditions presented a flat and polygonal morphology, with some thin filopodia protruding from the membrane surface in different directions, showing that both conditions allowed good cellular adhesion. Cell adhesion to the heterostructure surface was also confirmed through the analysis of the distribution of actin stress fibers after 3 days in culture. This analysis showed that the hOBs cytoplasm was crossed by well-defined stress fibers, most of them found in parallel orientation, an indicator of a structured cell disposition and complete adhesion (Figure 4c), in agreement with the SEM observations. These results are consistent with previous studies which have shown that P(VDF-TrFE) is biocompatible and facilitates good cell adhesion, as well as cell–material interactions.^27,37,57^

### 2.6 Effect of the Magnetic-Field-Induced Electrical Stimulation on Osteoblast Proliferation and Differentiation

**Figure 5:**
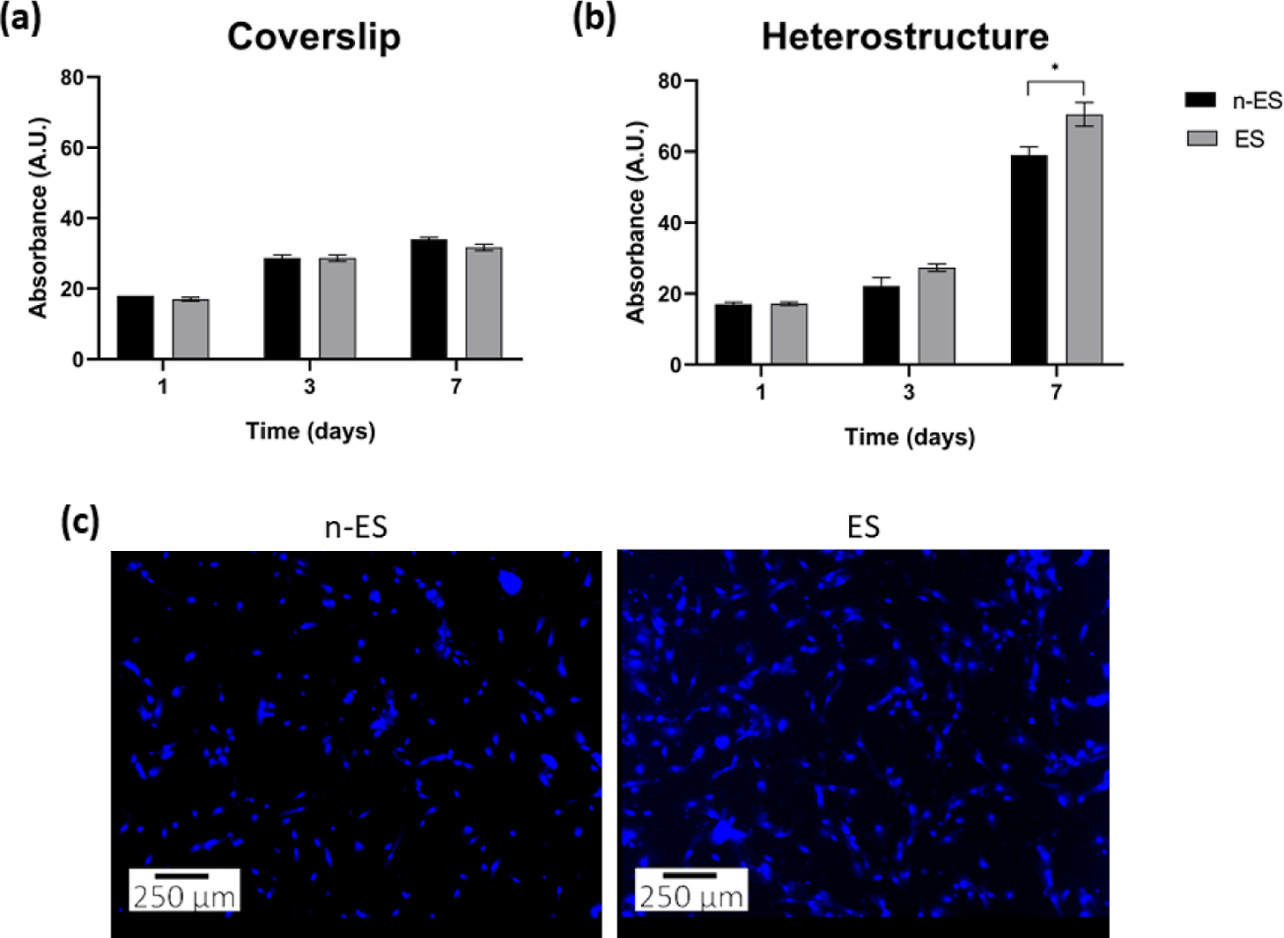
Proliferation of hOBs cultured on top of (a) glass coverslips or (b) FeGa/P(VDF-TrFE) heterostructures under magnetic-field-induced electrical stimulation (ES) and without it (n-ES) for 1, 3, and 7 days in culture. No significant differences were found when growing on the glass coverslip at any time-points analyzed, but statistically significant differences were found at 7 days in osteoblasts grown on the heterostructures. Asterisks indicate significant differences (*p* < 0.05) among both conditions at the same time-point. (c) Images of cell nuclei (blue) of hOBs grown on top of the FeGa/P(VDF-TrFE) heterostructures in the two conditions at day 7. ES was started 24 h after cell seeding (day 1).

Previous studies have suggested that external magnetic stimulation can induce additional physical effects on cells.^58^ Accordingly, to verify that the magnetic field used in our study did not impact cell proliferation, we conducted a control cell culture experiment. Osteoblasts were cultured on glass coverslips, excluding the presence of magnetoelectric layers, and exposed (or not exposed) to the same daily magnetoelectric stimulation regime for 7 days. When using glass coverslips under the applied magnetic field conditions, no discernible effects on cell proliferation were observed, neither in stimulated (ES) nor the nonstimulated (n-ES) hOBs cells (Figure 5a). The results demonstrate that this stimulation had neither detrimental nor beneficial effects on the cells, thus confirming the innocuous/neutral nature of the magnetic actuation under these parameters (400 Oe@100 Hz for 1 h daily).

After confirming that remote stimulation of osteoblasts does not compromise cell adhesion or viability and that the applied magnetic field does not interfere with the osteoblast proliferation when they are cultured on glass coverslips, we proceeded to analyze the effects of magnetic-field-induced electrical stimulation on osteoblast proliferation. Note that during the seeding of the cells and the 24 h adhesion period, the magnetic field was not applied, and consequently, the electric field on the heterostructures was zero. This analysis was conducted at 1, 3, and 7 days after osteoblast seeding on top of the magnetoelectric heterostructure. Under both conditions, the number of cells significantly increased over time. Interestingly, significant differences were observed between stimulated and nonstimulated heterostructures after 7 days in culture (Figure 5b). Namely, the number of cells was higher when the FeGa/P(VDF-TrFE) heterostructures were electrically actuated with the magnetic field, as confirmed by the images of the nuclei of the cells on day 7 (Figure 5c).

In addition to proliferation, electrical stimulation has also been described to enhance osteoblast differentiation. Osteoblast differentiation is usually divided in three stages: (i) cell proliferation, (ii) extracellular matrix (ECM) maturation, and (iii) ECM mineralization.^59,60^ During the proliferation phase, several ECM proteins, such as collagen I (COLI) and fibronectin, can be detected. The matrix maturation phase is characterized by the expression of specific genes needed for the synthesis and maturation of the ECM, like alkaline phosphatase (*ALPL*), *COLI*, and bone sialoprotein (*IBSP*). Finally, during matrix mineralization, genes encoding proteins such as osteocalcin (*BGLAP*) and osteonectin (*SPARC*) are expressed. Once mineralization is completed, calcium deposition can be visualized using different staining methods.

The ability of the magnetic-field-induced electrical stimulation to enhance the differentiation of hOBs grown on top of the FeGa/P(VDF-TrFE) heterostructures was assessed by analyzing the gene expression of several early and late osteoblast marker genes, production of both early and late osteoblast protein markers, activity of alkaline phosphatase (ALP), and formation of extracellular calcium deposits. Gene expression was analyzed through the whole culture period (proliferation and differentiation phases), whereas protein expression, ALP activity, and calcium deposits were only analyzed during the differentiation phase (7 and 14 days).

**Figure 6:**
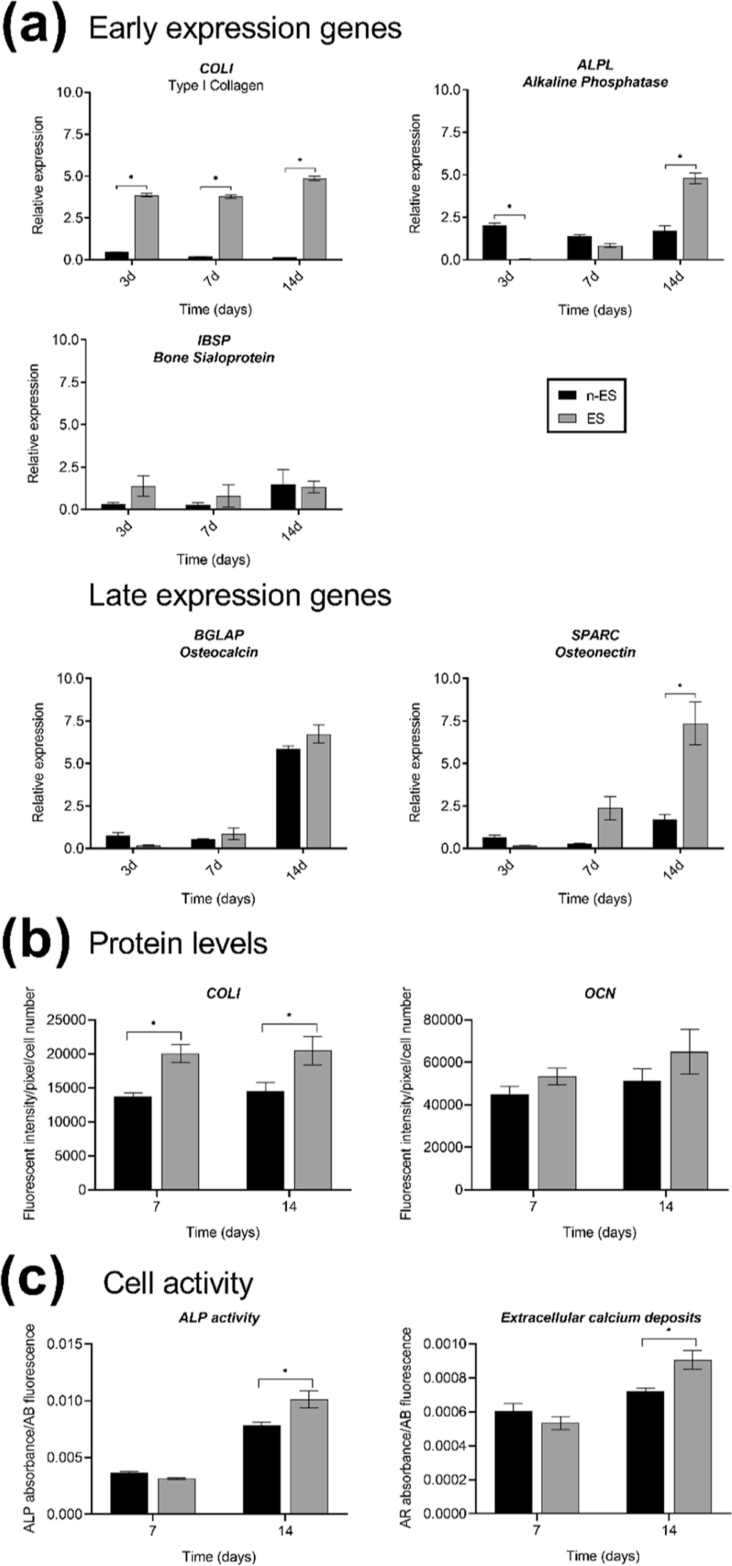
Differentiation of osteoblasts cultured on top of the FeGa/P(VDF-TrFE) heterostructures under magnetic-field-induced electrical stimulation (ES) and without stimulation (n-ES) for 3, 7, and 14 days. (a) Expression of osteoblast differentiation marker genes COLI, ALPL, IBSP, BGLAP, and SPARC. The proteins encoded by these genes are indicated below the names of the genes. The mRNA levels are expressed as the ratio of the target gene expression to that of the reference genes (TBP and HPRT1). (b) Relative fluorescent intensity of the COLI and the osteocalcin (OCN) osteoblast differentiation protein; (c) ALP activity and extracellular calcium deposits of the ECM. Asterisks indicate significant differences (*p* < 0.05) among the conditions at the same time-point.

First, we analyzed the expression of the three early osteogenic marker genes *COLI*, *ALPL* and *IBSP*, and the two late osteogenic marker genes *BGLAP* and *SPARC* in hOBs after 3, 7, and 14 days in culture under both conditions (Figure 6a). When compared with unstimulated cells, expression levels of *COLI* were drastically higher at all time-points in stimulated cells (more than four times for each time-point), and those of *ALPL* and *SPARC* were significantly increased only after 14 days of culture (more than 2.5-fold and more than 4-fold, respectively). Due to the electrical stimulation, the initial expression of ALPL during proliferation was nearly nonexistent but increased during matrix maturation. For the other two genes (*IBSP* and *BGLAP*), expression levels were similar at all time-points between stimulated and nonstimulated cells.

Next, protein levels were assessed after 7 and 14 days in culture through immunofluorescence detection of COLI, characteristic of the first stages of osteoblast differentiation and ECM maturation, and osteocalcin (OCN), a protein that is more abundant during the mineralization phase. COLI protein levels were significantly higher in stimulated than in nonstimulated hOBs at both time-points, whereas the levels of the OCN were similar (Figure 6b). These results are in agreement with the gene expression results.

In addition, ALP activity was quantified after 7 and 14 days in culture. After 7 days, no significant differences were observed between the two conditions tested. However, after 14 days, ALP activity strongly increased in both conditions, being significantly higher in the stimulated cells (Figure 6c).

Finally, to demonstrate that hOBs showed a mature differentiation state in the presence of the magnetoelectric stimulation, the capacity to mineralize the ECM was analyzed after 7 and 14 days in culture. A significant increase in extracellular calcium deposits was detected in the stimulated cells at day 14, but not at day 7 (Figure 6c).

The four experiments performed to quantify the osteoblast differentiation (gene expression, protein detection, ALP activity, and calcium deposits quantification) were all concordant and demonstrated that stimulation using the flexible magnetoelectric heterostructures significantly increased and possibly accelerated hOBs differentiation.

As demonstrated by the magnetoelectric characterization (Figure 2d,e), when a magnetic field is applied to the FeGa/P(VDF-TrFE) heterostructures, an electric field is generated. This generated electric field should be capable of stimulating osteoblast cells (Scheme 1), consequently promoting cell proliferation and differentiation.^8,9^

The proposed mechanism of this electrical field enhanced proliferation and differentiation is based on the insights of different authors. It is suggested that the proximity of the electric field to the plasma membrane triggers the opening of voltage-gated calcium channels. This, in turn, allows a controlled influx of calcium ions into the cells, activating signaling pathways such as the calcium–calmodulin pathway, ultimately contributing to bone regeneration.^8,9^ Within this framework, the ES condition would also trigger the opening of stretch-activated calcium channels and activate membrane receptors coupled to phospholipase C enzyme, which is involved in calcium release from the reticulum calcium storages.^61,62^

In previous studies where a piezoelectric material was used to electrically stimulate osteoblast, we demonstrated that when osteoblast adhere to a network of ZnO nanosheets^21^ or to a PVDF scaffold,^37^ the resulting adhesion forces caused the ZnO nanosheets or the PVDF fibers to bend, generating a local electric field of sufficient magnitude to stimulate the cells and modulate their activity. In both studies, calcium content within the cells was monitored over time, revealing high- or low-amplitude Ca^2+^ transients that can be correlated with the opening of voltage or stretch calcium channels (VGCCs or SACCs), or with the reorganization of plasma membrane receptors, respectively. These findings suggest that a similar mechanistic process may also occur when using the FeGa/P(VDF-TrFE) heterostructure since the upper layer of this heterostructure is piezoelectric and thus it induces an electrical field when a magnetic field is applied.

Note that several authors have described an increase in cell proliferation when cells are remotely subjected to an electrical stimulus using different approaches based on piezoelectric/ferroelectric materials. For example, an increase in proliferation has been observed when using different piezoelectric layers activated by ultrasounds^63,64^ or different magnetoelectric systems activated magnetically.^27−29^ In addition, Liu et al.^29^ have analyzed osteoblast differentiation using magnetoelectric stimulation, reporting an enhanced differentiation of bone cells exposed to 2300 Oe DC magnetic field during 12 h daily for 14 days. In fact, when compared with other studies using magnetoelectric approaches, the conditions used in our study (400 Oe@100 Hz, for 1 h daily) are rather mild. Studies using considerably longer times, higher fields or higher frequencies can be found in the literature.^22−24,30^

The good performance of the kapton/FeGa/P(VDF-TrFE) heterostructures probably stems from the planar interface between the magnetostrictive and the ferroelectric layers, which maximizes the strain transfer between the FeGa and the P(VDF-TrFE), and should, in turn, produce larger electric fields compared with those of other types of structures. Second, the flexible character of the kapton substrate should also play a key role in the enhanced hOBs proliferation and differentiation. Namely, common magnetoelectric systems grown on rigid substrates are prone to clamping effects,^65^ where the mechanical attachment of the magnetoelectric layers to the substrate impedes the proper transmission of the strain between the magnetostrictive and ferroelectric layers, thus hampering the production of electric fields. Although there are several strategies to minimize clamping effects,^44,66,67^ growing the magnetoelectric layers on flexible kapton layer appears to be an efficient and relatively straightforward strategy to reduce the clamping effect, and consequently induce sufficient electric fields to stimulate hOBs. Another factor that may have a positive influence on hOBs is the stimulation frequency. In our case, we used 100 Hz, which is commensurate with the typical response time of many of the cell processes.^68^ Note that the magnetoelectric heterostructures did not present any obvious sign of deterioration after the biological experiments.

In the long term, after in vivo studies, given its flexibility, this type of heterostructure might be suitable as a remotely actuated conformal graft for bone regeneration (in a “band-aid”-like approach).^69−71^

## 3 Conclusions

In this work, the potential use of flexible magnetoelectric heterostructures [i.e., kapton/FeGa/P(VDF-TrFE)] to promote cell proliferation, ECM maturation, and mineralization has been demonstrated. After characterizing the magnetic and ferroelectric properties of FeGa and P(VDF-TrFE) separately, evidence for strain-mediated magnetoelectric coupling between the two layers was obtained by PFM. The flexible character of the substrate, minimizing clamping effects, is probably crucial to induce such a coupling. Next, hOBs have been cultured onto the magnetoelectric heterostructures, after a prior treatment with O_2_ plasma to enhance hydrophilicity, and thus cell adhesion. By subjecting the whole structure (material + cells) to an alternating magnetic field (daily for a duration of 1 h), clear evidence of efficient magnetoelectric cell stimulation is obtained. The results may have an impact in advanced healthcare technologies based on wireless electrical cell stimulation, not only to accelerate the healing of bone fractures but also in other areas, such as muscle stimulation or neural tissue engineering.

## 4 Materials and Methods

### 4.1 Fabrication of the FeGa/P(VDF-TrFE) Heterostructures

Fe_72_Ga_28_ (at. %) alloy films (100 nm-thick) were grown by DC sputtering using Fe_75_Ga_25_ targets onto kapton foils (50 μm thick, HN 200 from Isovolta S.A.U.) previously metallized with a 10 nm thick sputtered Ti adhesion layer. Sputtering of both layers was performed using an AJA International magnetron system with a base pressure of 1 × 10^–8^ Torr and setting the working pressure at 3 × 10^–3^ Torr. The P(VDF-TrFE) copolymer top layer (250 nm thick) was grown by spin coating. P(VDF-TrFE) powders in a 70/30 wt ratio were first dissolved in diethyl ketone to form a transparent solution with a concentration of 4 wt/vol %. The solution was then spin-coated at 3000 rpm onto the sputtered FeGa film, for 60 s, using a Suss Microtech spinner. These conditions were selected based on our previous studies (see Figure S2) to obtain relatively thick P(VDF-TrFE) films without compromising their roughness. This facilitates the characterization of the structure and the ferroelectric properties of the polymer. Then, the samples were annealed at 125 °C for 6 h in air using a DZF-6020-ZZKD oven to promote the crystallization of the ferroelectric β-phase of P(VDF-TrFE). In order to increase the hydrophilicity of the naturally hydrophobic surface of P(VDF-TrFE), the samples were treated for 1 min with oxygen plasma using the PlasmaPro Cobra 100 ICP Etching System (HF 5 W, ICP 300 W, 17 mTorr, O_2_—50 sccm).

### 4.2 Structural and Morphological Characterization

The morphology of the different layers was investigated by SEM using a Zeiss MERLIN field emission SEM at 5 keV. The elemental composition was assessed by EDX using SEM with an acceleration voltage of 20 keV. The XRD patterns were acquired on a Philips X’Pert diffractometer using Cu Kα radiation in θ–2θ geometry. FTIR spectroscopy was recorded using a Hyperion 2000 microspectrometer (Bruker) with an attenuated total reflectance (ATR) objective and a mercury–cadmium–telluride (MCT) detector cooled with liquid nitrogen. The spectra were registered for 132 scans with a 4 cm^–1^ resolution.

### 4.3 Magnetic Characterization

Magnetic hysteresis loops were recorded at room temperature along in-plane and perpendicular-to-plane directions using a vibrating sample magnetometer (VSM) from MicroSense.

### 4.4 Atomic Force Microscopy and Piezo-Response Force Microscopy Characterization

All AFM measurements were conducted on an MFP-3D Asylum AFM (Asylum Research, Oxford Instruments). The cantilevers used for PFM experiments were PPP-EFM tips (Nano sensors; Schaffhausen, Switzerland) with a stiffness constant of *k* = 2 N/m and coated with Ptlr. PFM lithography was done by applying −9 V on a scan area of 5 × 5 μm^2^ and +9 V on a scan area of 2.5 × 2.5 μm^2^ through the tip. The tip was in contact with the sample applying a force of 60 nN. To record the ferroelectric hysteresis loops, we used the same cantilevers and the same applied force.

### 4.5 Contact Angle Measurements

Contact angle measurements were conducted to assess the hydrophilicity of the films using the sessile-drop technique (DSA 100 system from Krüss). Microdroplets of 10 μL of milli-Q water were employed for such measurements. Contact angle values were obtained by averaging several measurements in different locations on the films’ surfaces.

### 4.6 Magnetic Stimulation Setup

**Figure 7:**
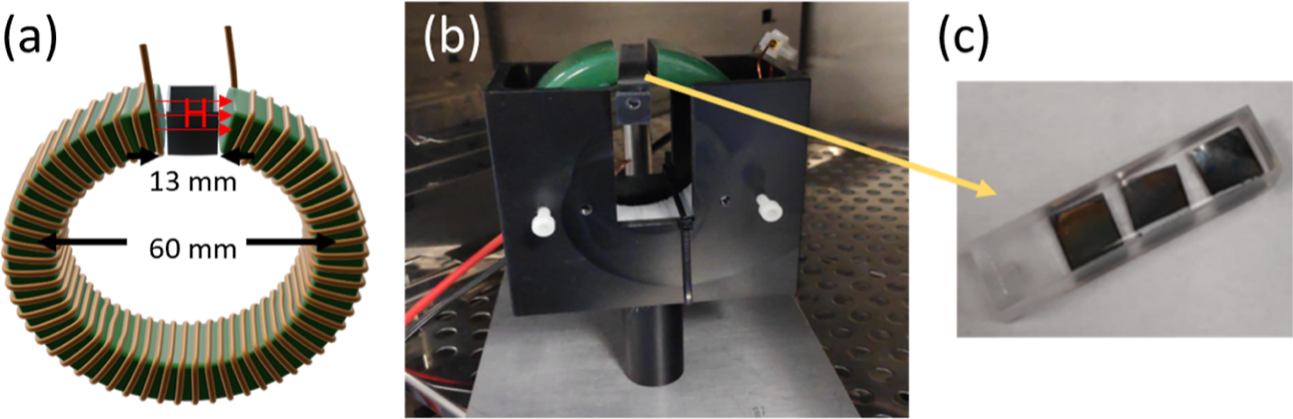
(a) Schematic representation of the toroidal actuation setup; (b) photograph of the experimental system. (c) Photograph of the cuvettes.

The magnetic actuation device used to stimulate the cells consisted of a ferrite toroid (60 mm in diameter with a 13 mm gap), with 65 turns of 1 mm laminated copper wire wound around it. The actuator was powered by a Siglent SDG-1025 signal generator and an Accel TS200 power supply and was able to generate magnetic field in the gap of the electromagnet ranging between 100 Oe (@110 kHz) and 1000 Oe (@1 Hz). In the current experiments, a field of 400 Oe at 100 Hz was used. The samples were placed in the gap of the electromagnet by using a homemade poly methyl-methacrylate (PMMA) cuvette (Figure 7).

### 4.7 Cell Culture and Stimulation Conditions

hOBs were isolated from explanted trabecular bone obtained from a healthy donor after a removal surgery with informed consent. The procedure to obtain primary cell cultures from human bones was approved by the Ethics Committee (Comissió d’Ètica en Experimentació Animal i Humana) of the Universitat Autònoma de Barcelona (CCEAH-2672). The isolation procedure was performed according to the method detailed by Gallagher.^72^ Briefly, the trabecular bone was isolated from the cortex and connective tissues. Then, the trabecular bone fragments were washed in phosphate-buffered saline (PBS) and cut into pieces of 3–5 mm in diameter. Finally, bone fragments were vortex-mixed to remove hematopoietic cells. The bone fragments were cultured as explants in 75 cm^2^ flasks for at least 14 days. hOBS were used because they are considered to have a physiological phenotype of an osteoblast differentiating into an osteocyte.^73^

The bone explants, as well as the isolated hOBs, were cultured in Dulbecco’s modified Eagles medium (DMEM; Gibco, Thermo Fisher Scientific, Waltham, MA, USA) supplemented with 20% fetal bovine serum (FBS; Gibco) and 2% penicillin–streptomycin (P/S; Biowest, Riverside, MO, USA), under standard conditions (37 °C, 5% CO_2_).

The experiments were performed by using hOBs between passages 2 and 10. Cells were maintained at 37 °C in a humidified atmosphere of 5% CO_2_ (standard conditions). The FeGa/P(VDF-TrFE) heterostructures were sterilized using a UV-C led sterilization chamber (59S, Shenzhen, China) and individually introduced into a four-well plate. Then, different numbers of cells, according to the experiment (see the following sections), were seeded into each well. Cells were cultured with DMEM supplemented with 20% FBS and 2% P/S for 24 h to allow cell adhesion to the heterostructures. Afterward, the heterostructures with the adhered hOBs were transferred to the cuvettes and cultured daily either under a magnetic field of 400 Oe at 100 Hz for 1 h to induce an electrical stimulation (electrically stimulated; ES) or without magnetic field (not electrically stimulated; n-ES).

For viability, morphology, adhesion, and proliferation experiments, cells were cultured with DMEM supplemented with 20% FBS and 2% P/S. For the differentiation experiments (gene expression, osteogenic protein markers immunodetection, quantification of ALP activity, and mineralization), the serum content was reduced to 10% FBS.

### 4.8 Cell Viability Assay

Cell viability was assessed by the detection of the intracellular esterase’s activity using the live/dead viability/cytotoxicity kit for mammalian cells (Invitrogen, Thermo Fisher Scientific, Waltham, MA, USA) according to the manufacturer’s protocol. To perform the assay, 20,000 cells were seeded on the heterostructures and cultured for 3 days under ES and n-ES conditions. The cells were incubated with the kit reagents for 15 min and immediately observed. Live cells showed green color because their esterase activity converts nonfluorescent calcein AM into fluorescent, whereas dead cells showed red color because of the permeability of their damaged plasma membrane to propidium iodide. Samples were observed at 40× magnification with an Olympus IX71 Inverted Fluorescence Microscope (Olympus, Shinjuku, Japan) equipped with a 10MP CMOS Camera. Representative images from different regions of each sample were captured, and the percentage of viable cells of a minimum of 2500 cells in five fields was calculated for each condition.

### 4.9 Cell Morphology Analysis

After the cell viability assay, the same samples were processed to be analyzed by SEM. Briefly, cells were washed in 0.1 M cacodylate buffer saline (CBS), fixed in 2.5% glutaraldehyde 0.1 M in CBS for 45 min at room temperature (RT) and rinsed again twice in CBS. Cell dehydration was performed in a series of increasing ethanol concentrations (50, 70, 90, and twice 100%) for 8 min each. Finally, the samples were dried using hexamethyldisilazane (Electron Microscopy Sciences, Hatfield, PA, USA) for 15 min. The samples were then mounted on special stubs and metalized by using a E5000 Sputter Coater (Emitech, France) for 2 min. Finally, they were analyzed using a MERLIN FE-SEM (Zeiss, Oberkochen, Germany) in order to observe cell morphology at different magnifications. Representative images from different regions of each culture sample were obtained.

### 4.10 Actin Cytoskeleton Distribution

The analysis of the cell cytoskeleton was performed by staining of the actin filaments. In these studies, 20,000 cells were seeded onto the heterostructures, and after 72 h, the samples were washed twice in PBS and cells fixed in 4% paraformaldehyde (PFA) in PBS for 20 min at RT. After washing again twice in PBS, the cells were permeabilized with 1% Tween-20 in PBS for 20 min. The samples were then incubated with a mixture of Phalloidin-Atto 488 (Sigma-Aldrich, Merck, Burlington, MA, USA), and Hoechst 33258 (Sigma-Aldrich) for 60 min in the dark at RT. Finally, cells were washed in PBS, air-dried, and mounted on 35 mm glass bottom dishes (Ibidi, Gräfelfing, Germany) using ProLong Antifade mounting solution (Life Technologies, Carlsbad, CA, USA). The cytoskeleton evaluation was done at 630× in a Confocal TCS SP5 (Leica, Heerbrugg, Switzerland) by using a PL APO 63× objective. Representative images from different regions of each sample were obtained.

### 4.11 Cell Proliferation Assay

The proliferation of hOBs was determined by the quantification of the cell activity using alamar blue reagent (Thermo Fisher Scientific) at days 1, 3, and 7 of culture. Briefly, 20,000 cells were seeded into each well of a four-well plate containing the heterostructure. After 24 h to allow cell adhesion (day 1), heterostructures with adhered cells were moved to a new well to discard cells growing outside them. Then, fresh DMEM without phenol red (Gibco) and with 10% alamar blue was added. Cells were incubated for 4 h in the dark and under standard conditions. After incubation, the supernatant was collected, and its fluorescence was measured at a wavelength of 585 nm after excitation at 560 nm on a Spark multimode microplate reader (Tecan, Männedorf, Switzerland). After supernatant collection, samples were transferred to the plastic buckets, and fresh medium was added to the cultures for the assay to be repeated at 3 and 7 days under ES or n-ES conditions. The experiments were performed in triplicate. After the last test at day 7, the samples were washed twice in PBS and the cells were fixed in 4% PFA in PBS for 20 min at RT. After washing again twice in PBS, cells were incubated with Hoechst 33258 (Sigma-Aldrich) for 15 min in the dark at RT. Finally, cells were washed in PBS and observed at 40× magnification with an Olympus IX71 Inverted Fluorescence Microscope equipped with a 10MP CMOS Camera. Representative images from different regions of each culture sample were obtained.

### 4.12 Expression of Osteogenic Marker Genes

The expression of osteogenic marker genes encoding type I collagen (*COLI*), alkaline phosphatase (*ALPL*), bone sialoprotein (*IBSP*), osteocalcin (*BGLAP*), and osteonectin (*SPARC*) was analyzed by real-time quantitative polymerase chain reaction (qPCR). For gene expression analysis, 100,000 hOBs were seeded onto the heterostructures and the total RNA was extracted from the cell cultures at 3, 7, and 14 days using the Maxwell RSC simplyRNA tissue kit (Promega, Madison, WI, USA) according to the manufacturer’s protocol. Then, the RNA concentration and purity were determined using a Nanodrop spectrophotometer (Nanodrop 1000, Thermo Scientific, Thermo Fisher Scientific). Reverse transcription was performed with 500 ng of total RNA using the iScript cDNA synthesis kit (BioRad, Hercules, CA, USA) according to the manufacturer’s instructions. The mRNA levels were assayed in triplicate in CFX384 arrays (BioRad) using 5 μL of iTaq Universal SYBR Green Supermix (BioRad), 0.5 μL of PrimePCR Assays (BioRad), and 20 ng of cDNA in a total volume of 10 μL. Then, a PCR amplification was performed following the next steps: initial heating at 95 °C for 3 min, followed by 40 cycles at 95 °C for 10 s, 60 °C for 30 s, and a final melt curve from 65 to 95 °C, in 0.5 °C increment each 5 s in a C1000 Touch Thermal Cycler (BioRad). The expression values were obtained from cycle quantification (Cq) values determined with BioRad CFX Maestro Software. The target gene levels are expressed as a relative value: the ratio of the target gene expression to that of the reference TATA-box binding protein (*TBP*) and hypoxanthine phosphoribosyl transferase (*HPRT1*) genes. The relative gene expression was calculated as 2-ΔCq. Validated PrimePCR SYBR Green Assays (BioRad) for *COLI* (qHsaCED0043248), *ALPL* (qHsaCID0010031), *IBSP* (qHsaCED0002933), *BGLAP* (qHsaCED0038437), *SPARC* (qHsaCID0010332), *TBP* (qHsaCID0007122), and *HPRT1* (qHsaCID0016375) were used.

### 4.13 Immunodetection of Osteogenic Markers

Quantification of the osteogenic markers was performed by immunofluorescence detection of two different proteins involved in osteoblast differentiation: type I collagen (COLI) as an early differentiation marker and osteocalcin (OCN) as a late differentiation marker. To perform immunodetection, 100,000 hOBs were seeded onto the heterostructures and cultured for 7 and 14 days in both ES and n-ES conditions. Then, the cells were fixed in 4% PFA in PBS for 20 min at RT, permeabilized with PBS containing 1% Tween-20 for 20 min, and blocked with 5% bovine serum albumin (BSA; Sigma) in PBS for 20 min at RT. The samples were then incubated overnight at 4 °C with primary antibodies: rabbit antiosteocalcin T-4743 (1:200; Peninsula Laboratories, San Carlos, CA, USA) or mouse monoclonal anticollagen I LB-1197 (1:200; Cosmo Bio, Tokyo, Japan). After being rinsed twice with 1% BSA in PBS, the cells were incubated for 1 h at RT with the secondary antibody Alexa Fluor594-conjugated goat antirabbit (Thermo Fisher Scientific) or Alexa Fluor 488-conjugated goat antimouse (Thermo Fisher Scientific) diluted 1:400 in PBS together with the nuclear dye Hoechst 33258 (Sigma-Aldrich). Images from randomly selected regions were obtained, and the fluorescence intensity was measured. To measure fluorescence intensity, at least five different captures from each time-point and condition were taken with the same exposure time, laser power, and detector voltage and analyzed using FIJI (ver. 1.53u, 2022, available from http://imagej.nih.gov/ij). In each 8 bit image from the specific protein channel, the integrated density (sum of pixel values in a selected area multiplied by the escalated area of one pixel, where the selected area is determined by using a threshold) normalized to the number of cells (number of cell nuclei on the blue channel) was calculated.

### 4.14 Quantification of the ALP Activity

The differentiation of hOBs was also studied by measuring the ALP activity. For this assay, 100,000 cells were seeded onto the heterostructures and cultured during 7 and 14 days. Then, the ALP activity was determined by the hydrolysis of *p*-nitrophenyl phosphate (pNPP), which produces *p*-nitro-phenol (pNP). Briefly, 300 μL of 1-step pNPP (Thermo Fisher Scientific) was added to the cells and, after 30 min incubation at RT, the supernatant was collected. The absorbance was measured at 405 nm using a Spark multimode microplate reader (Tecan) and normalized using Alamar Blue results performed on the same samples prior to ALP. Three independent experiments were used for each time-point and condition.

### 4.15 Mineralization Assay

The mineralization of the ECM by differentiated osteoblasts was evaluated through the detection of calcium deposits. Specifically, calcium deposition was determined by alizarin red staining (ARS, Sigma-Aldrich). Briefly, 100,000 hOBs were seeded on the heterostructures for 7 and 14 days. Later, the cells were rinsed twice in PBS, fixed in 4% PFA in PBS for 20 min at RT and then washed twice with distilled water. Then, the samples were incubated with 2% ARS in distilled water at pH 4.2 for 45 min. Finally, the samples were washed four times with distilled water to clear any nonbound alizarin red. To measure the absorbance, the incorporated ARS dye was extracted from the cell cultures with 10% cetylpiridinium chloride (CPC, Sigma-Aldrich) in 10 mM sodium phosphate (Fluka, Honeywell, Charlotte, NC, USA) at pH 7 for 15 min on a shaker at RT. The extracted product was transferred to a 96-well plate, and the absorbance was measured at 540 nm using a Spark multimode microplate reader (Tecan) and normalized using alamar blue results performed on the same samples prior to ARS. The experiments were performed in triplicate.

### 4.16 Statistical Analysis

All quantitative data were analyzed with GraphPad Prism 8 (GraphPad Software Inc., San Diego, CA, USA) and presented as the mean ± standard error of the mean. Statistical comparisons were performed using one-way analysis of variance (ANOVA) with Bonferroni correction for multiple comparison tests for cell proliferation, qPCR assays, osteogenic protein markers intensity, ALP activity, and ARS absorbance. A value of *p* < 0.05 was considered to be significant.

## Data Availability

The data sets and Supporting Information can be acquired online or by contacting the corresponding authors.

## Supporting Information Available

Photograph of a piece of kapton/FeGa/P(VDF-TrFE) sample during manual bending and dependence of the thickness and roughness of the P(VDF-TrFE) films depending on synthesis conditions (PDF)
