# Supplementary material for: Enhanced Proliferation and Differentiation of Human Osteoblasts by Remotely Controlled Magnetic-Field-Induced Electric Stimulation Using Flexible Substrates
Source: ACS Appl Mater Interfaces. 2023 Dec 5;15(50):58054–66. doi: 10.1021/acsami.3c09428 (PMC10739596; doi:10.1021/acsami.3c09428)
Supplement: Supplementary file 1 — am3c09428_si_001.pdf [file am3c09428_si_001.pdf]

SUPPORTING INFORMATION

Enhanced proliferation and differentiation of human osteoblasts by remotely controlled magnetic-field-induced electric stimulation using flexible substrates

Oriol Careta[a], Aliona Nicolenco[b,c], Filippos Perdikos[d], Andreu Blanquer[a,] Elena Ibañez[a], Eva Pellicer[b], Christina Stefani[b], Borja Sepúlveda[e], Josep Nogués[d,f], Jordi Sort[b,f,], Carme Nogués[a,*]*

[a] Departament de Biologia Cel·lular, Fisiologia i Immunologia, Universitat Autònoma de Barcelona, E-08193, Bellaterra (Cerdanyola del Vallès), Spain. Email: carme.nogues@uab.cat

[b] Departament de Física, Universitat Autònoma de Barcelona, E-08193, Bellaterra (Cerdanyola del Vallès), Spain. Email: jordi.sort@uab.cat

[c] CIDETEC, Parque Científico y Tecnológico de Gipuzkoa, Paseo Miramón, 191, San Sebastián, Spain

[d] Catalan Institute of Nanoscience and Nanotechnology (ICN2), CSIC and BIST, Campus UAB, Bellaterra, E-08193 Barcelona, Spain

[e] Instituto de Microelectronica de Barcelona (IMB-CNM, CSIC), Campus UAB, E-08193, Bellaterra, Barcelona, Spain

[f] Institució Catalana de Recerca i Estudis Avançats (ICREA), Pg. Lluís Companys 23, E-08010, Barcelona, Spain

[*]Corresponding authors

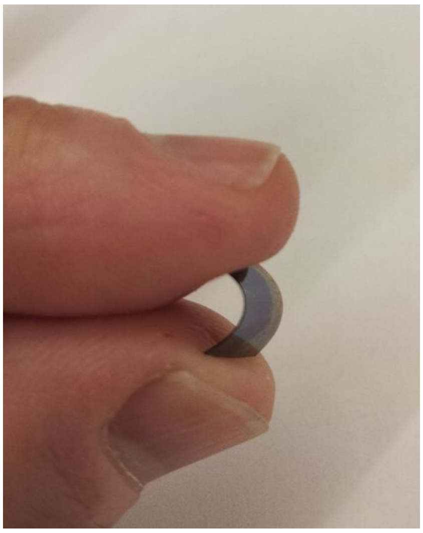

Figure S1: Photograph taken on a piece of Kapton/FeGa/P(VDF-TrFE) sample during manual bending, to show that the sample is flexible, and the layers remain well adhered to the substrate (Kapton) during mechanical straining.

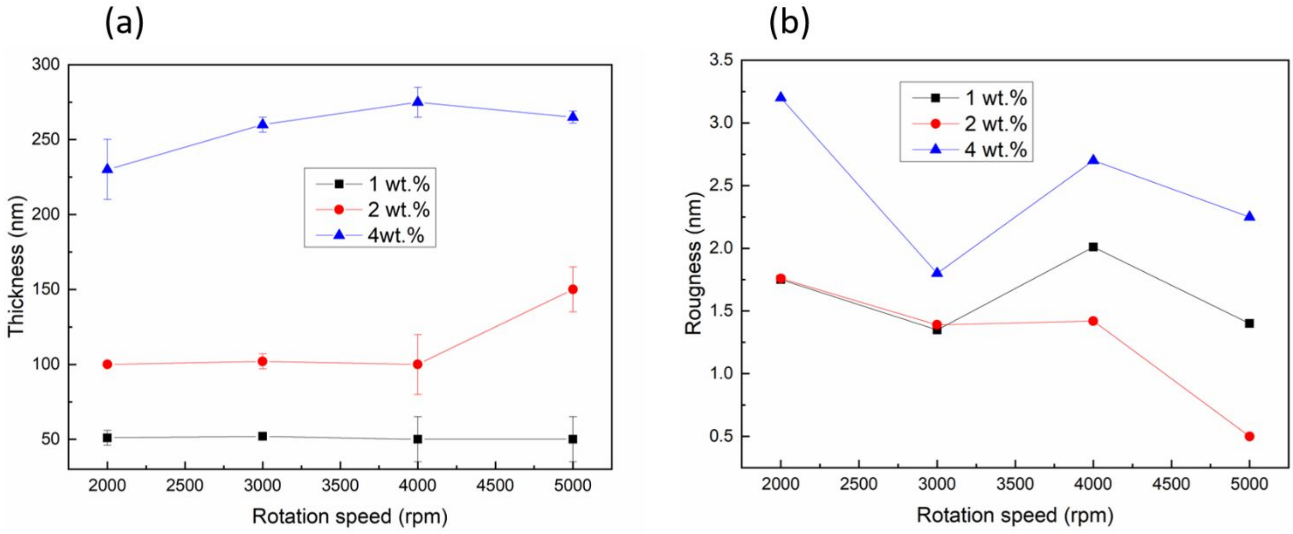

Figure S2: Dependence of the thickness and roughness of the P(VDF-TrFE) films depending on the angular velocity during spin coating and the concentration of P(VDF-TrFE) powders in the solution.
